# Construction and efficacy evaluation of a prognostic assessment model for corticosteroid treatment in patients with sudden sensorineural hearing loss based on lipid metabolism profile

**DOI:** 10.3389/fneur.2026.1769720

**Published:** 2026-03-18

**Authors:** Gaoqing Luo, Qinghua Lin, Chenglong Xiao, Jingyi Zheng

**Affiliations:** Department of Otolaryngology, Fujian Provincial Governmental Hospital, Fuzhou, Fujian, China

**Keywords:** corticosteroid treatment, lipid metabolism, nomogram, prognosis, sudden sensorineural hearing loss

## Abstract

**Objective:**

To explore the correlation between lipid metabolism profile, clinical indicators and prognosis of corticosteroid treatment in sudden sensorineural hearing loss (SSNHL) patients, and construct/verify a prognostic assessment model based on lipid metabolism profile for clinical individualized treatment.

**Methods:**

A retrospective study enrolled 446 SSNHL patients (divided into training set, *n* = 312; validation set, *n* = 134) who received corticosteroid treatment. Patients were grouped into good/poor prognosis groups by 4-week post-treatment pure tone average (PTA) improvement. Differences in indicators were compared; multivariate logistic regression identified independent factors to build a nomogram. Calibration curves, decision curve analysis (DCA) and ROC curves evaluated the model.

**Results:**

Poor prognosis group had higher age, diabetes/hypertension rates, ApoB/ApoB/ApoA ratio, non-HDL-C, disease duration, total deafness rate, and lower HDL-C/ApoA (all *p* < 0.05). Independent factors: older age, diabetes, higher ApoB/ApoA ratio, longer disease duration (risk factors), higher HDL-C (protective factor, all *p* < 0.05). The nomogram showed good consistency (Hosmer-Lemeshow *p* = 0.960/0.472), predictive efficacy (AUC = 0.785/0.744) and clinical net benefits in both sets.

**Conclusion:**

Age, diabetes, HDL-C, ApoB/ApoA ratio and disease duration are key factors for SSNHL corticosteroid treatment prognosis. The nomogram based on these indicators has reliable predictive efficacy, serving as an effective tool for clinical prognosis assessment and individualized treatment.

## 1 Introduction

Sudden sensorineural hearing loss (SSNHL) is a type of sensorineural hearing impairment with unknown etiology. Its core feature is sudden hearing loss of unknown cause within 72 h, with a hearing threshold decrease of ≥20 dB HL in at least two adjacent frequencies. Some patients are accompanied by tinnitus, aural fullness, or vertigo. The incidence is approximately 5–20 per 100,000 people and shows an increasing trend year by year (1, 2). As an emergency in otolaryngology, delayed treatment or poor prognosis of SSNHL may lead to permanent hearing damage, seriously affecting patients’ language communication ability and quality of life. Therefore, improving prognosis and enhancing treatment accuracy are core goals of clinical research (3).

Currently, corticosteroid treatment is the first-line standard regimen for SSNHL, including systemic oral methylprednisolone or intratympanic dexamethasone injection. It exerts therapeutic effects by anti-inflammation, reducing inner ear edema, and improving microcirculation (4, 5). However, clinical practice shows that the prognosis of corticosteroid treatment in SSNHL patients varies significantly: approximately 30–50% of patients have no obvious hearing improvement or even ineffective treatment after therapy, which is closely related to patients’ individual characteristics, underlying diseases, and pathophysiological status (6). Therefore, screening key prognostic factors and constructing reliable prognostic assessment tools are of great significance for identifying high-risk patients and adjusting individualized treatment strategies.

Previous studies have confirmed that age, history of diabetes, and disease duration are important clinical factors affecting the prognosis of SSNHL: elderly patients have degenerative changes in inner ear hair cells and nerve function, resulting in weak repair ability; patients with diabetes are prone to vascular endothelial injury, which impairs inner ear microcirculation; for patients with disease duration exceeding 7 days, the duration of inner ear ischemia and hypoxia is prolonged, increasing the risk of irreversible damage (7–9). In addition, lipid metabolism disorders are closely associated with vascular diseases, and inner ear microcirculatory disturbance is considered one of the important pathogenesis of SSNHL, suggesting that lipid metabolism indicators may be involved in prognostic regulation. However, existing studies mostly focus on conventional lipid metabolism indicators, and the results are controversial (10, 11). At the same time, there is a lack of quantitative prognostic models integrating lipid metabolism indicators and clinical indicators. Clinical assessment mostly relies on subjective experience, making it difficult to achieve accurate prognostic judgment.

Based on the above research status, this retrospective study analyzed the clinical data of SSNHL patients who received standardized corticosteroid treatment, focusing on exploring the correlation between lipid metabolism profile and the prognosis of corticosteroid treatment. Independent influencing factors were screened using multivariate logistic regression, and a visual nomogram prediction model was constructed. This study aims to fill the gap in the application of lipid metabolism indicators in SSNHL prognostic assessment, provide an objective and convenient prognostic assessment tool for clinical practice, further guide individualized treatment, and improve patients’ treatment outcomes.

## 2 Materials and methods

### 2.1 Study subjects and grouping criteria

This was an analytical, observational, open-label retrospective study. The study designer, data collector, and statistical analyst were independent of each other, and the data collector and statistical analyst were blinded to the study design. Patients with unilateral SSNHL who received corticosteroid treatment in our hospital from January 2020 to May 2025were enrolled.

#### 2.1.1 Diagnostic criteria for SSNHL

Referenced to the Clinical Practice Guideline: Sudden Hearing Loss by AAO-HNSF (2019): (1) Hearing loss occurs within ≤72 h, with a pure tone threshold decrease of ≥20 dB HL in at least two adjacent frequencies (0.5/1/2 kHz or 1/2/4 kHz); (2) Other diseases that may cause sudden hearing loss are excluded by temporal bone CT, inner ear MRI, vestibular function examination, etc.; (3) May be accompanied by tinnitus, aural fullness, vertigo, or balance disorders, but these are not essential for diagnosis (12).

#### 2.1.2 Inclusion criteria

(1) Meets the above diagnostic criteria for SSNHL; (2) Aged 18–75 years; (3) Receives standardized corticosteroid treatment (systemic oral or intratympanic injection) with a complete treatment plan, no premature withdrawal, drug replacement, or dose adjustment; (4) Complete clinical data, including traceable pre-treatment general clinical data, lipid metabolism indicator test reports, and pure tone hearing test records; (5) Completes follow-up reexamination at 4 weeks after treatment, with clear prognostic outcome.

#### 2.1.3 Exclusion criteria

(1) Complicated with other ear diseases such as chronic suppurative otitis media, middle ear cholesteatoma, acoustic neuroma, or Ménière’s disease; (2) Presence of contraindications to corticosteroid treatment, such as active tuberculosis, uncontrolled severe hypertension, uncontrolled diabetes, active peptic ulcer, severe osteoporosis, active stage of autoimmune diseases (e.g., systemic lupus erythematosus), etc.; (3) Use of lipid-lowering drugs (statins, fibrates), glucocorticoids outside the study protocol, oral contraceptives, or thyroid hormones within 3 months; (4) Complicated with severe organ dysfunction; (5) Complicated with malignant tumors, acute myocardial infarction/cerebral infarction, or mental illness that prevents cooperation with follow-up; (6) Pregnant or lactating women; (7) Lack of pre-treatment lipid metabolism indicators or post-treatment 4-week hearing reexamination data, or loss to follow-up or death during follow-up that makes prognosis undetermined.

Sample size was estimated with reference to previous studies on the prognosis of corticosteroid treatment in SSNHL patients using PASS 15.0 software. The significance level *α* = 0.05 (two-tailed), power (1-*β*) = 0.80, and allowable error *δ* = 10% (i.e., 10% of the expected effect size) were set. The sample size formula for binary data was used: $n=\frac{\left[Z_{\alpha /2}\sqrt{2P(1-P)}+Z_{\beta }\sqrt{P_{0}(1-P_{0})+P_{1}(1-P_{1})}\right]}{{\left(P_{1}-P_{0}\right)}^{2}}$ . Among them, P0 = 0.35 and P1 = 0.385, and the minimum sample size was calculated to be 380 cases. Considering a data loss rate of approximately 15% in retrospective studies, an additional 57 cases were added, and the final sample size was determined to be no less than 437 cases.

A total of 446 eligible SSNHL patients were finally included in this study. Simple random sampling was used to divide the patients into a training set and a validation set at a ratio of 7:3. The specific process was as follows: A unique serial number was assigned to each eligible patient, and the sample() function in R 4.2.1 software was used to generate random numbers with a fixed random seed (seed = 123) to ensure reproducibility of the grouping results. Patients with random numbers ranked in the top 70% were assigned to the training set (*n* = 312), and the remaining 30% were assigned to the validation set (*n* = 134).

All patients received standardized corticosteroid treatment. The treatment regimen was selected based on the presence of systemic hormone contraindications, and the basic adjuvant treatments were consistent across all patients: (1) Microcirculation-improving agents: *Ginkgo biloba* extract injection (20 mL, once daily intravenously) or oral *Ginkgo biloba* extract tablets (40 mg, three times daily); (2) Neurotrophic agents: Methylcobalamin injection (1 mg, once daily intramuscularly) or oral methylcobalamin tablets (0.5 mg, three times daily). The choice of administration route (intravenous/intramuscular vs. oral) was based on patient tolerance, with no between-group differences in the type or dosage of adjuvant drugs; (3) Systemic oral hormone (for patients without contraindications): Methylprednisolone injection at a dose of 40 mg once daily, administered intravenously for 5 consecutive days; no dose reduction was required after the 5-day treatment, and the subsequent period until the 4th week of follow-up was supplemented with basic adjuvant treatments to maintain therapeutic continuity, with the total course of treatment (including adjuvant therapy) being 4 weeks; (4) Intratympanic hormone injection (for patients with systemic hormone contraindications, or those with poor hearing improvement [PTA improvement <10 dB HL] after 5 days of intravenous methylprednisolone treatment): Dexamethasone injection (5 mg/mL). Patients received intratympanic injection starting from the 6th day after enrollment: after topical anesthesia (2% lidocaine ear drops), puncture was performed in the anteroinferior quadrant of the tympanic membrane, and 5 mg dexamethasone was injected. The patient maintained a position with the affected ear upward for 30 min; the injection was performed twice a week for 3 consecutive weeks (6 times in total), and hearing was reexamined 1 week after the last intratympanic injection (total course of combined treatment [systemic + intratympanic] was 4 weeks).

Treatment efficacy was determined according to the improvement degree of PTA at 4 weeks after treatment: (1) Cure: PTA returns to normal or pre-illness level; (2) Improvement: PTA decreases by ≥15 dB HL compared with pre-treatment but does not meet the cure standard; (3) Ineffective: PTA decreases by <15 dB HL compared with pre-treatment, or no hearing improvement/aggravation. Patients with cure or improvement were classified into the good prognosis group, and those with ineffective treatment were classified into the poor prognosis group.

This study only conducted retrospective statistical analysis of data from the previous in-hospital patient database. Patients’ names and contact information were kept confidential, and the ethics committee waived the requirement for a written application for this study.

### 2.2 Observed index

#### 2.2.1 General clinical data

General clinical data of patients were extracted from the hospital information system (HIS) and electronic medical record system with a missing rate of <1%. For the few missing data points, including gender (male/female), age, history of hypertension (previously diagnosed or blood pressure ≥140/90 mmHg at consultation), history of diabetes (previously diagnosed or fasting blood glucose ≥7.0 mmol/L), history of coronary heart disease (previously diagnosed or confirmed by coronary CTA), body mass index (BMI), and fibrinogen (Fib). For the few missing data points, multiple imputation was used to fill in the gaps based on the distribution of other related indicators. Finally, all included patients had complete general clinical data without missing key information that would affect the analysis.

#### 2.2.2 Detection of lipid metabolism indicators

Five milliliters of elbow venous blood was collected from patients on an empty stomach (8–12 h) 1 day before treatment, placed in a coagulation-promoting tube, left at room temperature for 30 min, and then centrifuged at 3000 r/min for 15 min (centrifugal radius 10 cm) to separate serum, which was immediately detected. A Mindray BS-2000 M automatic biochemical analyzer was used with original reagents from Hitachi. Indoor quality control was performed before detection. The detected indicators included total cholesterol (TC), triglycerides (TG), high-density lipoprotein cholesterol (HDL-C), low-density lipoprotein cholesterol (LDL-C, mmol/L), apolipoprotein A (ApoA), and apolipoprotein B (ApoB). ApoB/ApoA ratio and non-HDL-C (non-HDL-C = TC - HDL-C) were further calculated.

#### 2.2.3 Detection of hearing indicators

In a standard soundproof room (background noise ≤30 dB A), a pure tone audiometer of Otometrics brand (model 1,066) was used by certified hearing technicians, and the operation complied with *Acoustics - Determination of Pure Tone Air Conduction and Bone Conduction Thresholds* (GB/T 16403-1996). The affected ear (determined by chief complaint and audiogram, divided into left ear/right ear), disease duration (time from the onset of hearing loss to the first visit), PTA, and hearing curve type were recorded. PTA was calculated as the average of hearing thresholds at four frequencies (500, 1,000, 2000, 4,000 Hz). The hearing curve types were divided into 4 categories: (1) Rising type: Hearing threshold decrease ≥20 dB HL at low frequencies (0.25, 0.5 kHz), normal or <10 dB HL decrease at medium and high frequencies (1, 2, 4, 8 kHz); (2) Descending type: Hearing threshold decrease ≥20 dB HL at high frequencies (2, 4, 8 kHz), normal or <10 dB HL decrease at low frequencies (0.25, 0.5 kHz); (3) Flat type: Similar degree of hearing threshold decrease at all frequencies (0.25–8 kHz), with a difference between the maximum and minimum hearing thresholds <20 dB HL; (4) Total deafness type: Hearing threshold ≥90 dB HL at all detected frequencies.

### 2.3 Statistical analyses

SPSS 26.0 software and R 4.2.1 software were used for statistical analysis. For measurement data, normality was tested using the Shapiro–Wilk test. A *p* value > 0.05 was considered to indicate a normal distribution. Data conforming to normal distribution were expressed as mean ± standard deviation (x̅±s), and independent samples t-test was used for comparison between two groups; measurement data not conforming to normal distribution were expressed as median (interquartile range) [M (Q1, Q3)], and Mann–Whitney U test was used for comparison between two groups. Count data were expressed as number (percentage) [*n* (%)], and chi-square test was used for comparison between groups; Fisher’s exact test was used when the theoretical frequency was less than 5.

Factors with *p* < 0.05 in univariate analysis were included in multivariate logistic regression analysis, and forward stepwise regression method was used to identify independent influencing factors associated with the prognosis of corticosteroid treatment. Based on the results of multivariate logistic regression analysis, a nomogram prediction model was constructed using the rms package in R software. Bootstrap resampling method (1,000 repetitions) was used for internal validation of the model in the training set, and external validation was performed in the validation set.

Calibration curves were plotted to observe the consistency between predicted probability and actual occurrence probability; DCA was performed to analyze the net benefit of the model under different threshold probabilities; ROC curves were plotted and AUC was calculated to evaluate the predictive efficacy of the model (a larger AUC indicates stronger predictive ability of the model). A *p* value <0.05 was considered statistically significant.

## 3 Results

### 3.1 Comparison of general clinical data between training set and validation set

There were no statistically significant differences in general clinical data (gender, age, history of hypertension, history of diabetes, history of coronary heart disease, BMI, Fib) between the training set and validation set (all *p* > 0.05, Table 1), indicating that the clinical data of patients in the two datasets were comparable and could be used for subsequent research and validation.

**Table 1:** Comparison of general clinical data between training set and validation set [(*x_*±*s*), *M* (*Q_1_*, *Q_3_*), *n* (%)].

General clinical data	Training set (*n* = 312)	Validation set (*n* = 134)	*t/Z/χ^2^*	*P*
Gender			0.984	0.321
Male	151 (48.4)	58 (43.3)		
Female	161 (51.6)	76 (56.7)		
Age (years)	60 (46, 70)	59 (47, 69)	0.175	0.861
History of hypertension	111 (35.6)	44 (32.8)	0.311	0.577
History of diabetes	67 (21.5)	26 (19.4)	0.244	0.622
History of coronary heart disease	10 (3.2)	4 (3.0)	0.015	0.903
BMI (kg/m^2^)	23.88 ± 3.51	23.61 ± 3.36	0.696	0.487
Fib (g/L)	2.83 (2.41, 3.25)	2.77 (2.37, 3.20)	0.861	0.586

BMI, Body mass index; Fib, Fibrinogen.

### 3.2 Comparison of general clinical data between poor prognosis group and good prognosis group in the training set

Among the 312 SSNHL patients in the training set, 91 cases (29.2%) were cured, 132 cases (42.3%) showed improvement, and 89 cases (28.5%) were ineffective after 4 weeks of standardized corticosteroid treatment. Patients with cure or improvement were included in the good prognosis group (*n* = 223, 71.5%), and those with ineffective treatment were included in the poor prognosis group (*n* = 89, 28.5%). The poor prognosis group had higher age, higher proportions of hypertension history and diabetes history than the good prognosis group (all *p* < 0.05). There were no statistically significant differences in gender, history of coronary heart disease, BMI, or Fib between the two groups (all *p* > 0.05, Table 2).

**Table 2:** Comparison of general clinical data between poor prognosis group and good prognosis group in the training set [(*x_*±*s*), *M* (*Q_1_*, *Q_3_*), *n* (%)].

General clinical data	Poor prognosis group (*n* = 89)	Good prognosis group (*n* = 223)	*t/Z/χ^2^*	*P*
Gender			0.001	0.985
Male	43 (48.3)	108 (48.4)		
Female	46 (51.7)	115 (51.6)		
Age (years)	67 (55, 72)	57 (42, 68)	4.921	<0.001
History of hypertension	42 (47.2)	69 (30.9)	7.328	0.007
History of diabetes	32 (36.0)	35 (15.7)	15.484	<0.001
History of coronary heart disease	1 (1.1)	9 (4.0)	1.739	0.187
BMI (kg/m^2^)	24.50 ± 3.51	23.65 ± 3.49	1.708	0.089
Fib (g/L)	2.85 (2.42, 3.37)	2.81 (2.40, 3.17)	1.074	0.283

BMI, Body mass index; Fib, Fibrinogen.

### 3.3 Comparison of lipid metabolism indicators between poor prognosis group and good prognosis group in the training set

The poor prognosis group had higher levels of ApoB, ApoB/ApoA ratio, and non-HDL-C, and lower levels of HDL-C and ApoA than the good prognosis group (all *p* < 0.05). There were no statistically significant differences in TC, TG, or LDL-C between the two groups (all *p* > 0.05, Table 3).

**Table 3:** Comparison of lipid metabolism indicators between poor prognosis group and good prognosis group in the training set [(*x_*±*s*), *M* (*Q_1_*, *Q_3_*)].

Lipid metabolism indicator	Poor prognosis group (*n* = 89)	Good prognosis group (*n* = 223)	*t/Z*	*P*
TC (mmol/L)	5.03 ± 1.18	5.14 ± 1.15	0.760	0.448
TG (mmol/L)	0.89 (0.61, 1.27)	0.84 (0.61, 1.17)	0.263	0.793
HDL-C (mmol/L)	1.35 (1.09, 1.62)	1.49 (1.28, 1.76)	3.636	<0.001
LDL-C (mmol/L)	3.12 ± 1.00	3.26 ± 1.01	1.057	0.291
ApoA (g/L)	1.23 ± 0.25	1.38 ± 0.24	5.888	<0.001
ApoB (g/L)	1.07 ± 0.25	0.97 ± 0.26	3.815	<0.001
ApoB/ApoA	0.88 (0.67, 1.07)	0.70 (0.57, 0.88)	4.858	<0.001
Non-HDL-C (mmol/L)	3.66 ± 1.09	3.60 ± 1.13	0.439	0.661

TC, Total cholesterol; TG, Triglycerides; HDL-C, High-density lipoprotein cholesterol; LDL-C, Low-density lipoprotein cholesterol; ApoA, Apolipoprotein A; ApoB, Apolipoprotein B.

### 3.4 Comparison of hearing indicators between poor prognosis group and good prognosis group in the training set

The poor prognosis group had longer disease duration and higher proportion of total deafness patients than the good prognosis group (all *p* < 0.05). There were no statistically significant differences in PTA at onset or distribution of affected ears between the two groups (all *p* > 0.05, Table 4).

**Table 4:** Comparison of hearing indicators between poor prognosis group and good prognosis group in the training set [(*x_*±*s*), *M* (*Q_1_*, *Q_3_*), *n* (%)].

Hearing indicator	Poor prognosis group (*n* = 89)	Good prognosis group (*n* = 223)	*t/Z/χ^2^*	*p*
Affected ear			0.008	0.927
Left	48 (53.9)	119 (53.4)		
Right	41 (46.1)	104 (46.6)		
PTA (dB HL)	48 (38, 65)	52 (33, 78)	0.330	0.741
Disease duration (d)	7 (4, 18)	5 (3, 10)	3.809	<0.001
Hearing curve type			16.577	0.001
Rising type	21 (23.6)	81 (36.3)		
Descending type	23 (25.8)	27 (12.2)		
Flat type	40 (45.0)	81 (36.3)		
Total deafness	5 (5.6)	34 (15.2)		

PTA, Pure tone average.

### 3.5 Risk factors for poor prognosis of corticosteroid treatment in SSNHL patients

The prognosis of corticosteroid treatment in SSNHL patients was taken as the dependent variable, and indicators with inter-group differences were taken as independent variables. The specific assignment methods are shown in Table 5. Multivariate logistic regression analysis showed that older age [OR (95%CI) = 1.048 (1.024–1.073)], comorbidity with diabetes [OR (95%CI) = 1.449 (1.045–2.009)], higher ApoB/ApoA ratio [OR (95%CI) = 3.578 (1.132–11.304)], and longer disease duration [OR (95%CI) = 1.060 (1.022–1.100)] were independent risk factors for poor prognosis of corticosteroid treatment in SSNHL patients, while higher HDL-C level [OR (95%CI) = 0.515 (0.281–0.945)] was a protective factor against poor prognosis (all *p* < 0.05, Table 6).

**Table 5:** Variable assignment methods.

Variable type	Variable name	Assignment method
Y (Dependent variable)	Prognosis	Good prognosis = 0; Poor prognosis = 1
X1 (Independent variable)	Age	Original measured value
X2 (Independent variable)	History of hypertension	With comorbidity = 1; Without comorbidity = 0
X3 (Independent variable)	History of diabetes	With comorbidity = 1; Without comorbidity = 0
X4 (Independent variable)	HDL-C	Original measured value
X5 (Independent variable)	ApoA	Original measured value
X6 (Independent variable)	ApoB	Original measured value
X7 (Independent variable)	ApoB/ApoA	Original measured value
X8 (Independent variable)	Disease duration	Original measured value
X9 (Independent variable)	Hearing curve type	No total deafness = 0; Total deafness = 1

**Table 6:** Multivariate logistic regression analysis of risk factors for poor prognosis of corticosteroid treatment in SSNHL patients.

Index	*β*	S. E.	Wald *χ^2^*	*P*	*OR*	Lower limit of 95%CI	Upper limit of 95%CI
Age	0.047	0.012	15.203	<0.001	1.048	1.024	1.073
Hypertension	0.021	0.324	0.004	0.948	1.021	0.542	1.926
Diabetes	0.371	0.167	4.948	0.027	1.449	1.045	2.009
HDL-C	−0.663	0.310	4.592	0.035	0.515	0.281	0.945
ApoA	0.129	2.410	0.003	0.957	1.138	0.010	128.063
ApoB	−2.980	2.749	1.175	0.278	0.051	0.000	11.114
ApoB/ApoA	1.275	0.587	4.716	0.030	3.578	1.132	11.304
Disease duration	0.058	0.019	9.666	0.002	1.060	1.022	1.100
Hearing curve type	−0.006	0.139	0.002	0.964	0.994	0.757	1.305
Constant	−7.231	3.199	5.108	0.024	0.001		

### 3.6 Nomogram model for predicting poor prognosis of corticosteroid treatment in SSNHL patients

**Figure 1:**
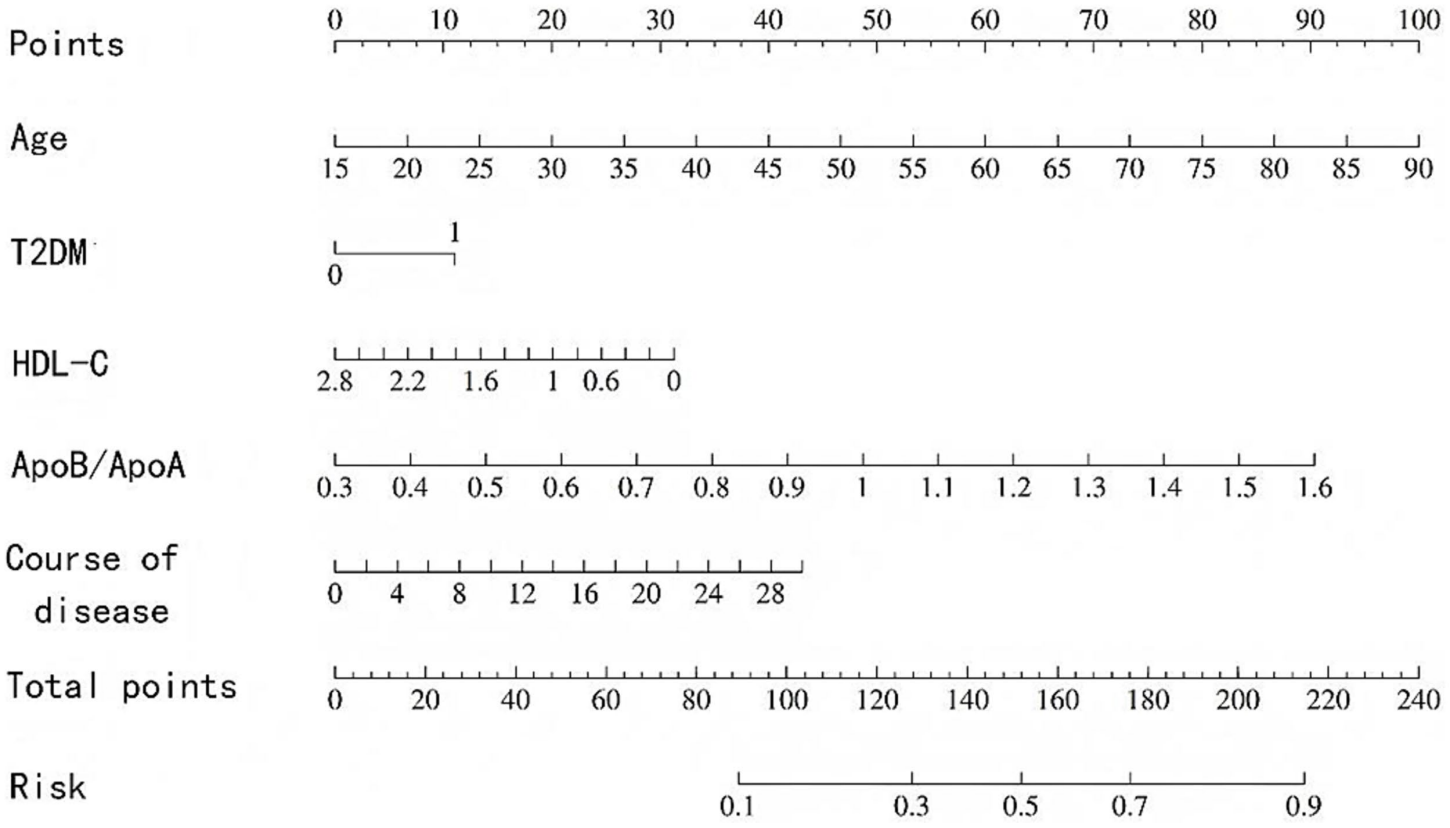
Nomogram model for predicting the risk of poor prognosis of corticosteroid treatment in SSNHL patients.

Based on the results of multivariate logistic regression analysis, a nomogram model for predicting the risk of poor prognosis of corticosteroid treatment in SSNHL patients was constructed using age, diabetes, HDL-C, ApoB/ApoA ratio, and disease duration (Figure 1). It can be intuitively observed from the nomogram that for SSNHL patients, older age, comorbidity with diabetes, higher ApoB/ApoA ratio, longer disease duration, and lower HDL-C level are associated with higher scores and probabilities of poor prognosis of corticosteroid treatment.

### 3.7 Fit of the nomogram model for predicting poor prognosis of corticosteroid treatment in SSNHL patients

**Figure 2:**
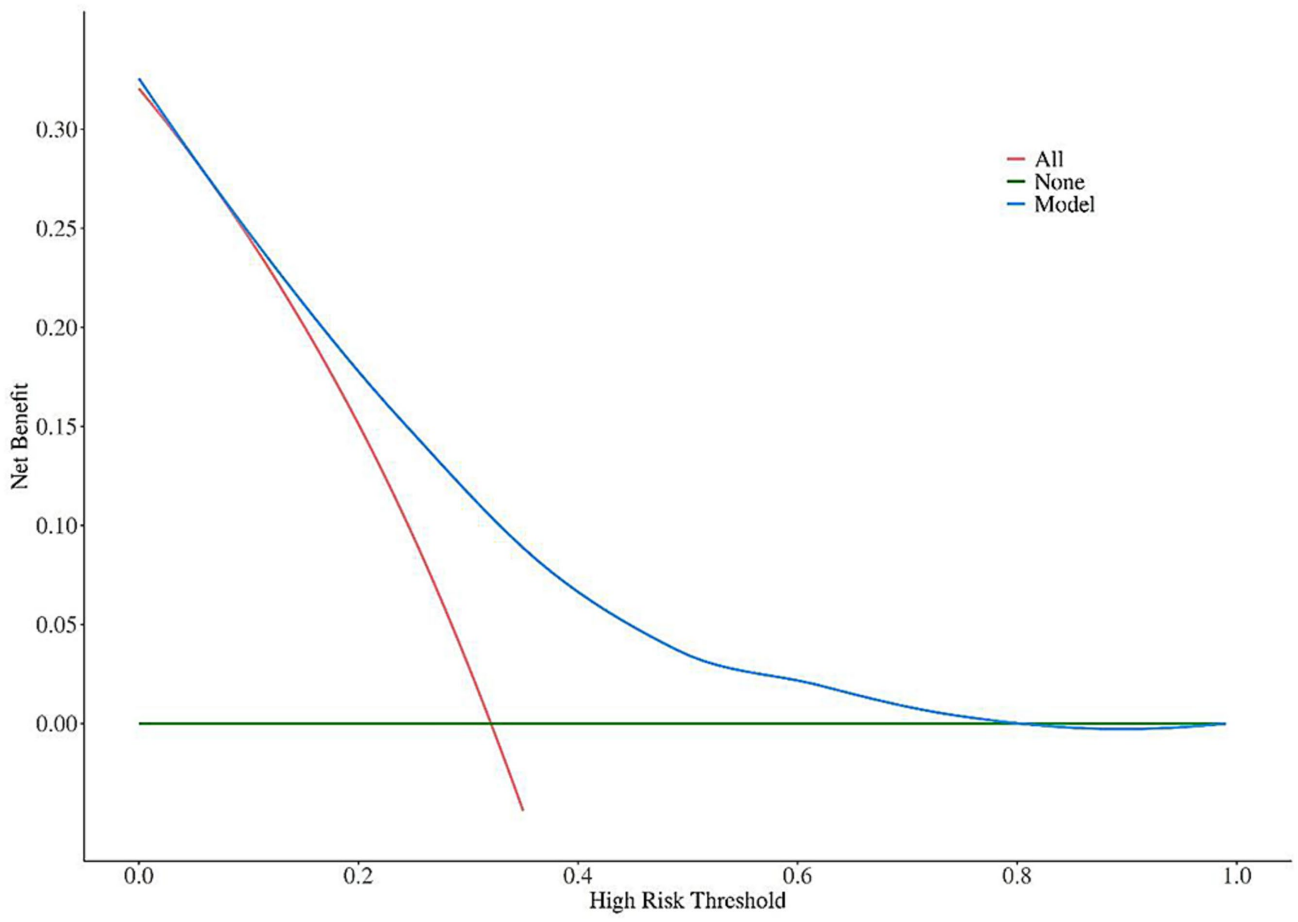
Calibration curve analysis of the nomogram model for predicting poor prognosis of corticosteroid treatment in SSNHL patients (training set).

**Figure 3:**
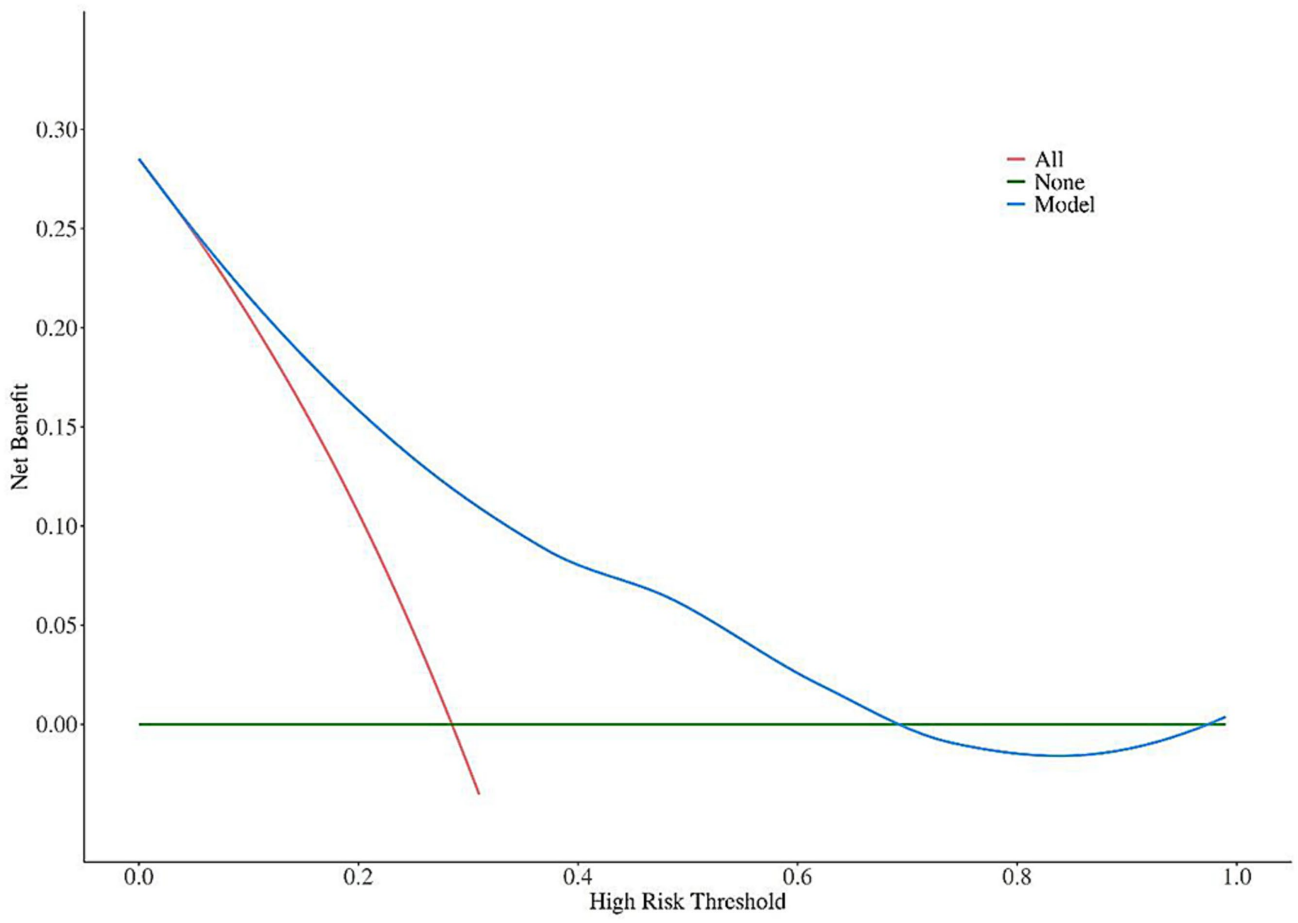
Calibration curve analysis of the nomogram model for predicting poor prognosis of corticosteroid treatment in SSNHL patients (validation set).

Calibration curves were used to evaluate the goodness of fit of the nomogram model in the training set and validation set. The results showed that in the training set, the Hosmer-Lemeshow χ^2^ = 2.528, *p* = 0.960; in the validation set, the Hosmer-Lemeshow χ^2^ = 7.611, *p* = 0.472, indicating that the model had high goodness of fit in both the training set and validation set (Figures 2, 3).

### 3.8 Predictive efficacy of the nomogram model for predicting poor prognosis of corticosteroid treatment in SSNHL patients

**Figure 4:**
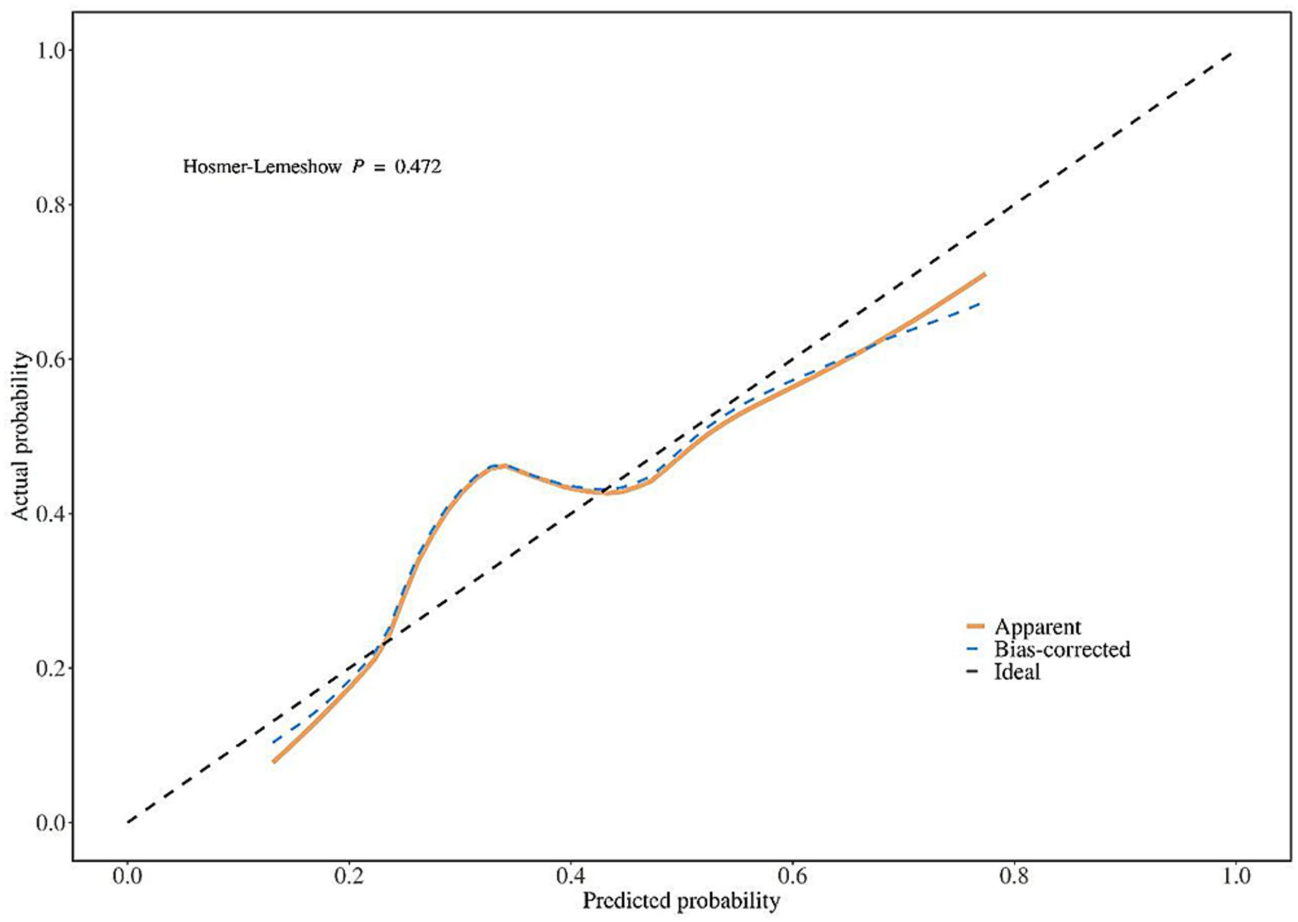
ROC curve analysis of the nomogram model for predicting poor prognosis of corticosteroid treatment in SSNHL patients (training set and validation set).

ROC curve analysis was used to evaluate the predictive efficacy of the nomogram model in the training set and validation set. The results showed that in the training set, the AUC (95%CI) of the model was 0.785 (0.730–0.840); in the validation set, the AUC (95%CI) was 0.744 (0.655–0.833), indicating that the model had high predictive efficacy in both the training set and validation set (Figure 4).

### 3.9 Clinical application value of the nomogram model for predicting poor prognosis of corticosteroid treatment in SSNHL patients

**Figure 5:**
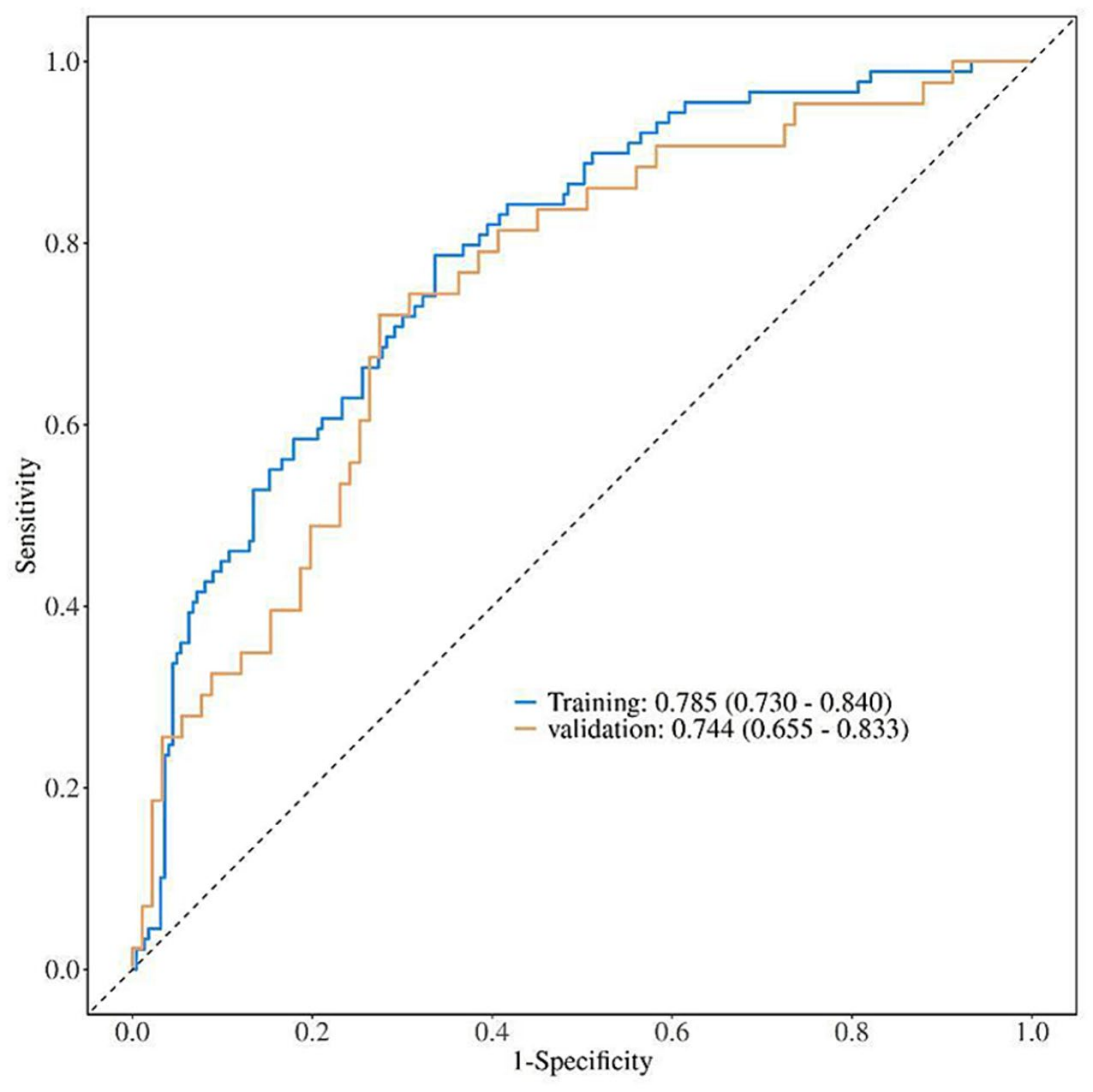
DCA curve analysis of the nomogram model for predicting poor prognosis of corticosteroid treatment in SSNHL patients (training set).

**Figure 6:**
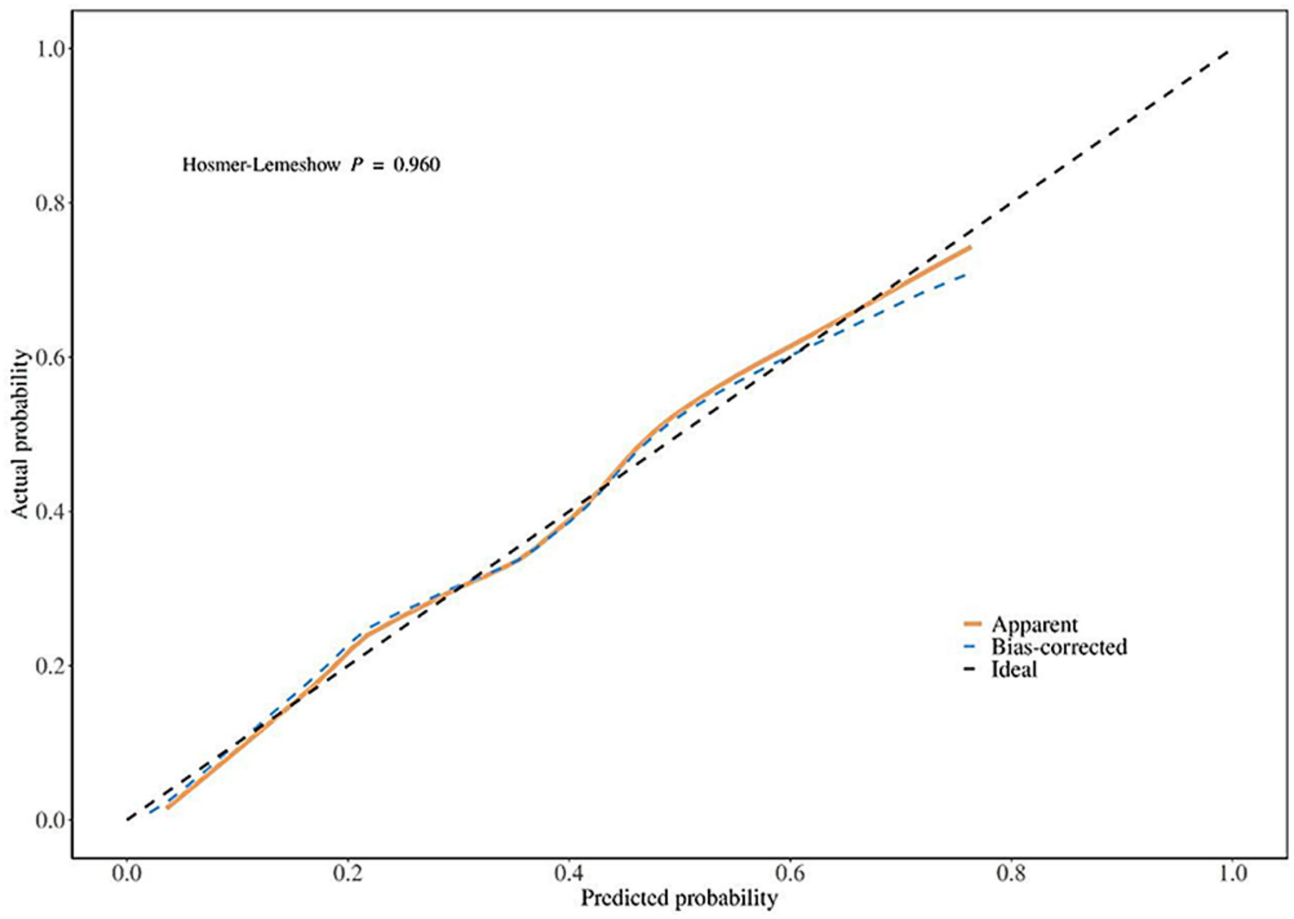
DCA curve analysis of the nomogram model for predicting poor prognosis of corticosteroid treatment in SSNHL patients (validation set).

DCA curves were used to analyze the clinical net benefit of the nomogram model in the training set and validation set. The results showed that in the training set, the model had a clinical net benefit >0 within the threshold probability range of 0.05–0.70; in the validation set, the model had a clinical net benefit >0 within the threshold probability range of 0.10–0.80, indicating that the model had high clinical application value in both the training set and validation set (Figures 5, 6).

## 4 Discussion

This study found that in the training set of SSNHL patients, the poor prognosis group had higher age, higher proportion of diabetes history, higher ApoB/ApoA ratio, and longer disease duration than the good prognosis group, while lower HDL-C and ApoA levels. Further multivariate logistic regression confirmed that older age, comorbidity with diabetes, increased ApoB/ApoA ratio, and prolonged disease duration were independent risk factors for poor prognosis of corticosteroid treatment in SSNHL patients, and increased HDL-C was a protective factor. The nomogram model constructed based on the above indicators showed excellent goodness of fit and good predictive efficacy in both the training set and validation set, and had significant clinical net benefits within specific threshold probability ranges.

The age of the poor prognosis group was significantly higher than that of the good prognosis group (*p* < 0.001), and the risk of poor prognosis increased by 4.8% for each 1-year increase in age. The core mechanism of this association is that degenerative changes in inner ear tissues and decreased hormone response efficiency in elderly patients are related to prognosis. From the perspective of tissue repair, hearing recovery in SSNHL depends on the functional repair of inner ear hair cells and spiral ganglion cells. However, the number of spiral ganglion cells in elderly patients decreases progressively with age, and the integrity of hair cell cilia bundles declines. Even though standardized corticosteroid treatment was used in this study to reduce acute inner ear inflammation and edema, it was difficult to reverse the already occurred nerve cell loss and hair cell structural damage (12, 13). In addition, the expression level of glucocorticoid receptors (GR) in elderly patients may decrease, leading to insufficient activation of the hormone anti-inflammatory pathway and ultimately weakening the therapeutic effect (14).

The proportion of patients with diabetes in the poor prognosis group (36.0%) was significantly higher than that in the good prognosis group (15.7%) in the training set, and the risk of poor prognosis increased by 44.9% in patients with diabetes. It should be noted that this study excluded patients with uncontrolled severe diabetes; therefore, the results better reflect the impact of long-term diabetes-induced organic damage to inner ear blood vessels on prognosis: even if blood glucose is stably controlled, the damage caused by previous hyperglycemia still continuously interferes with the effect of corticosteroid treatment (13). Long-term hyperglycemia can cause non-enzymatic glycation of the basement membrane of inner ear capillaries, leading to the accumulation of advanced glycation end products (AGEs), which thickens the basement membrane and narrows the lumen (15). In this study, whether oral hormone or intratympanic hormone injection was used, the drug needed to reach the injury site through the inner ear capillary wall. However, the thickened basement membrane forms a drug penetration barrier, resulting in insufficient local hormone concentration in the inner ear and directly reducing the anti-inflammatory effect (16). In addition, oxidative stress associated with diabetes activates inner ear microglia, which release proinflammatory factors that antagonize the anti-inflammatory effect of hormones. At the same time, it aggravates the damage to the auditory nerve myelin sheath, further offsetting the repair effect of neurotrophic drugs in the basic treatment of this study (17).

This study is the first to find in SSNHL prognostic research that conventional lipid metabolism indicators have no significant prognostic correlation, while lipoprotein functional indicators such as HDL-C and ApoB/ApoA ratio have clear predictive value. HDL-C can remove excess cholesterol from inner ear vascular endothelial cells through reverse cholesterol transport, avoiding lipid deposition (18); at the same time, it activates endothelial nitric oxide synthase (eNOS) to release NO, dilates inner ear capillaries, and improves perfusion, creating a good microenvironment for corticosteroid treatment (19). In this study, for each 0.1 mmol/L increase in HDL-C, the risk of poor prognosis decreased by approximately 8.5%, confirming the quantitative value of its protective effect. The risk factor effect of ApoB/ApoA ratio is essentially an imbalance in the anti-atherosclerotic vs. pro-atherosclerotic balance: ApoA is the core apolipoprotein of HDL, which directly determines the cholesterol clearance capacity of HDL (20); ApoB is the main apolipoprotein of LDL and a key carrier for lipid deposition (21). When the ApoB/ApoA ratio increases, the inner ear vascular endothelium faces the dual pressure of reduced clearance and increased deposition, which is prone to endothelial damage and weakens the improvement effect of hormones on microcirculation, ultimately leading to poor prognosis (22).

This study also found that the disease duration of the poor prognosis group in the training set was significantly longer than that of the good prognosis group, and the risk of poor prognosis increased by 6.0% for each 1-day prolongation of disease duration. This result is highly consistent with the clinical consensus of early treatment for SSNHL, and its core mechanism lies in the time-sensitive damage of inner ear cells to ischemia and hypoxia. In the early stage (disease duration ≤7 days), inner ear damage is mainly reversible edema and inflammation, with only mild deviation of hair cell cilia and nerve cells in a stress state. At this time, the hormone regimen in this study can quickly reduce edema, control inflammation, and restore inner ear perfusion with the assistance of microcirculation-improving drugs, leading to effective reversal of hearing damage; however, when the disease duration exceeds 7 days, continuous ischemia and hypoxia lead to hair cell cilia breakage and nerve cell apoptosis. Even if hormones can control subsequent inflammation, they cannot repair the already necrotic cells (23, 24). In addition, multivariate analysis in this study showed that hearing curve type had no significant prognostic correlation (*p* = 0.964), suggesting that the impact of disease duration on prognosis is independent of the degree of hearing damage. Even for mild hearing loss, if the disease duration exceeds 10 days, the risk of poor prognosis increases significantly, providing data support for the clinical recommendation that patients should seek medical attention as soon as possible regardless of the degree of damage.

Calibration curves showed that the nomogram model constructed based on the 5 independent influencing factors screened in this study had a high consistency between predicted probability and actual probability, with no obvious bias, and the AUC of ROC was >0.7, indicating that the model could effectively distinguish between patients with good and poor prognosis; DCA further confirmed that the net benefit of the model within the commonly used clinical threshold probability range was significantly higher than the strategies of “treat all” or “treat none,” which could avoid over-medical treatment and insufficient treatment. In addition, the visual design of the nomogram constructed in this study lowers the threshold for clinical application: doctors can quickly calculate the total score based on the patient’s age, history of diabetes, HDL-C level, ApoB/ApoA ratio, and disease duration, and then directly correspond to the risk of poor prognosis through the total score. For high-risk patients with a total score >120, the conventional oral hormone regimen can be adjusted to a combination of oral and intratympanic injection, or the dose of microcirculation-improving drugs can be increased; for low-risk patients with a total score <60, the conventional regimen is sufficient, which is conducive to reducing hormone-related adverse reactions.

However, this study is a single-center study, and all patients were from the same hospital, which may have regional selection bias. The relatively concentrated dietary structure and living habits of patients may lead to a higher proportion of lipid metabolism disorders, and the promotion of the model in other regions needs further verification; on the other hand, the hospital used a standardized corticosteroid treatment regimen, while different hospitals may have differences in hormone dose and administration frequency, and the efficacy of the model under non-standardized treatment regimens is still unclear. In addition, retrospective studies cannot completely avoid data loss bias. Although this study excluded patients with incomplete clinical data, unrecorded information in electronic medical records may have a potential impact on prognosis, and these factors were not included in the analysis. It should also be noted that this study used multivariate logistic regression to construct the prognostic model, which is a classic statistical method with clear interpretability but may have limitations in capturing complex non-linear relationships and interactions between variables. Recent studies have advocated the application of machine learning algorithms in hearing-related prognostic research, such as quadratic discriminant analysis and random forest, which can better handle high-dimensional data and improve prediction accuracy (25). In future work, we plan to incorporate these machine learning models to compare their predictive efficacy with the current logistic regression-based nomogram, and further optimize the model by integrating more potential predictors (e.g., inflammatory factors, genetic markers). This may help improve the accuracy and robustness of prognostic assessment for SSNHL patients receiving corticosteroid treatment.

## 5 Conclusion

In conclusion, this study confirms that increased age, comorbidity with diabetes, decreased HDL-C level, increased ApoB/ApoA ratio, and prolonged disease duration are independent risk factors for poor prognosis of corticosteroid treatment in SSNHL patients. The nomogram model constructed based on these indicators shows good goodness of fit, predictive efficacy, and clinical net benefit in both the training set and validation set.

## Data Availability

The original contributions presented in the study are included in the article/supplementary material, further inquiries can be directed to the corresponding author.
